# Exploring the Role of Genetic Variability and Lifestyle in Oxidative Stress Response for Healthy Aging and Longevity

**DOI:** 10.3390/ijms140816443

**Published:** 2013-08-08

**Authors:** Serena Dato, Paolina Crocco, Patrizia D’Aquila, Francesco de Rango, Dina Bellizzi, Giuseppina Rose, Giuseppe Passarino

**Affiliations:** Department of Biology, Ecology and Heart Science, University of Calabria, Ponte Pietro Bucci cubo 4c, Rende 87036, CS, Italy; E-Mails: s.dato@unical.it (S.D.); paolacrocco@tim.it (P.C.); d_patrizia2002@yahoo.it (P.D.); francesco.derango@unical.it (F.D.R.); dina.bellizzi@unical.it (D.B.); pinarose@unical.it (G.R.)

**Keywords:** oxidative stress response, aging, longevity, genetic variability, lifestyle

## Abstract

Oxidative stress is both the cause and consequence of impaired functional homeostasis characterizing human aging. The worsening efficiency of stress response with age represents a health risk and leads to the onset and accrual of major age-related diseases. In contrast, centenarians seem to have evolved conservative stress response mechanisms, probably derived from a combination of a diet rich in natural antioxidants, an active lifestyle and a favorable genetic background, particularly rich in genetic variants able to counteract the stress overload at the level of both nuclear and mitochondrial DNA. The integration of these factors could allow centenarians to maintain moderate levels of free radicals that exert beneficial signaling and modulator effects on cellular metabolism. Considering the hot debate on the efficacy of antioxidant supplementation in promoting healthy aging, in this review we gathered the existing information regarding genetic variability and lifestyle factors which potentially modulate the stress response at old age. Evidence reported here suggests that the integration of lifestyle factors (moderate physical activity and healthy nutrition) and genetic background could shift the balance in favor of the antioxidant cellular machinery by activating appropriate defense mechanisms in response to exceeding external and internal stress levels, and thus possibly achieving the prospect of living a longer life.

## 1. Introduction

The role of the oxidative stress response in healthy aging and longevity is a hot topic in the field of human aging studies. Comparisons among species with different rates of aging suggest that long-lived species tend to show reduced oxidative damage [1], reduced mitochondrial free radical production [2], increased antioxidant defenses [2], and increased resistance to oxidative stress both *in vivo* and *in vitro* [3]. However, a lack of correlation of oxidation with lifespan [4], or even an increase in oxidative damage/stress associated with long lifespan has also been reported [5]. The stress response is defined as the ability of initiating an array of regulatory processes in response to oxidative stress, including the activation of stress-gene expression and modification of stress-responsive signal transcription pathways. This stress response has been reported to be impaired with aging [6]. In humans, compelling evidence indicates that aging is associated with a general loss in oxidative stress tolerance [7]. Genetic modifications of the stress response with age have also been reported, with whole-genome transcriptional analyses of aged organisms and tissues demonstrating a general increase in the expression of genes involved in the stress responses [8]. Thus, it appears that the stress response to stress-induced cellular injury quantitatively increases with advancing age, but is less efficient. This failure to effectively respond to cellular challenges has been postulated to contribute to a reduction in stress tolerance and the development or worsening of the most relevant age-related diseases, ranging from degenerative [9,10] and cardiovascular diseases (CVD) [11,12] to rheumatoid arthritis [13], diabetes and cancer [14]. However, the molecular mechanisms activated by free radicals, involving the activation of a complex cascade of mediators acting at different levels, complicates the investigation of the above cascade in relation to age-related phenotypes. In particular, a clear comprehension of the complex relationship existing in an individual between constitutive risk/protective factors (genotype) for a poor/appropriate stress response and modifiable risk factors (*i.e.*, diet, environment and physical exercise), which could increase/decrease the level of oxidative stress, is still missing.

Aware of the difficulty of condensing the vast literature available on this topic in a single review, our aim is to underline the importance of genetic and non genetic factors in the aging organism’s ability to counteract the negative effects of oxidative stress and thus ultimately minimize health risks and possibly increase the individual’s possibilities of achieving a longer life.

### 1.1. Oxidative Stress at the Cellular Level

Oxidative stress is the consequence of an imbalance between the generation of oxidant molecules and the ability of endogenous anti-oxidant defense mechanisms to counteract it. Reactive Oxygen Species (ROS) are chemically reactive molecules containing oxygen that can be formed by external environmental sources such as radiation, ultraviolet light and pollution (including cigarette smoke) or as by-products of endogenous enzymatic activities, as in inflammation and more importantly in oxidative phosphorylation, the biochemical process by which energy released by oxidation of nutrients is converted into ATP (adenosine triphosphate) by mitochondria. In particular, during the first three steps of oxidative phosphorylation, electrons are transferred from electron donors to electron acceptors such as molecular oxygen. An insufficient reduction of oxygen, even by 2%–3%, gives rise to production of ROS and their reaction products that are also dangerously reactive. These ROS include the superoxide anion (O_2_^•^), hydroxyl radical (•HO), peroxyl radical (HOO•) as well as non-radical derivatives such as hydrogen peroxides (H_2_O_2_) and singlet oxygen (^1^Δ_g_O_2_) [15]. Free radicals can oxidize and damage nucleic acids, proteins and lipids, which are the major components of cell membranes. Because of the continuous generation of free radicals throughout cell life, mitochondria are key targets of the free radical attack.

Both in the nucleus and mitochondria, an increased baseline level of DNA oxidation has been associated with the age-related development of several cancers, such as breast, colon/rectum, and prostate cancers ([16] and references therein). Increased oxidation of RNA has been documented in diseases related to aging including dementia [17], Alzheimer’s [18] and Parkinson’s [19] diseases (AD and PD), atherosclerosis [20] and myopathies [21].

Furthermore, high amounts of nitric oxide (NO) and its derivative Reactive Nitrogen Species (RNS), such as peroxynitrite (ONOO^−^), a highly reactive radical produced by the reaction between NO and superoxide anion, have been observed to induce nitrosative modifications of proteins, lipids and nucleic acids, which in turn may influence cellular signaling pathways leading to cytotoxicity, neurodegeneration, and apoptotic cell death [22–24].

To counteract oxidative stress, cells have evolved sophisticated antioxidant machinery, composed of DNA repair enzymes and antioxidants, substances that inhibit or delay oxidation of substrates. Endogenous antioxidant defenses can be divided in non-enzymatic mechanisms (e.g., uric acid, glutathione, bilirubin, thiols, albumin, and nutritional factors, including vitamins and phenols) and enzymatic mechanisms [e.g., the superoxide dismutases (SOD), the glutathione peroxidases (GPx) and catalase (CAT)]. The efficient removal of superoxide free radicals maintains the integrity of membranes, reduces the risk of cancer, and slows down the aging process [15].

### 1.2. Oxidative Stress and Aging

A causative role of oxidative damage accumulation on aging, diseases and death was first proposed by Harman with the “Free Radical Theory of Aging” [25], and further extended in the 1970s to implicate the mitochondrial production of ROS [26]. This hypothesis stems from the evidence that cells using oxygen consequently generate ROS; increased ROS levels lead to a vicious cycle where ROS produced by the mitochondrial electron transport chain damages the mitochondria, leading exponentially to more ROS production and mitochondrial damage.

Normally, there is a balanced-equilibrium among bio-molecules, *i.e.*, between pro- and antioxidant elements. Although a physiological accumulation of radicals is normal, about 1% of ROS escape the control of the endogenous antioxidant systems daily [27]. With age, the excessive generation of free radicals, due to the progressive de-regulation of cellular metabolism, may overwhelm the natural cellular antioxidant scavenging response, generating a status of impaired homeostasis [28]. Thus, the worsening of the stress response with aging represents a major health risk, ultimately giving way to an increased general frailty of the organism, a condition of vulnerability which exposes elderly individuals to a higher risk of poor outcomes, including infections, disabilities, institutionalization, and death [29]. On the contrary, the “stress resistance paradigm” suggests that mechanisms conferring robustness to counteract multiple forms of stress can represent a potential avenue leading towards “healthy aging” [30].

It follows therefore that it should be sufficient to decrease ROS levels in order to increase the probability of healthy aging and live a longer life. However, recent findings claim that well studied mechanisms mediating longevity such as caloric restriction (CR), and specifically reduced glucose metabolism, in fact result in an increase in ROS accumulation by stimulating the basal metabolic rate, and consequently increase oxidative stress [31]. Accordingly, the consistent reduction of free radicals by antioxidant therapy was shown to be detrimental to healthy aging [32]. The reason for this contrasting role of ROS in aging is attributed to the induction of a hormetic response, *i.e.*, an adaptive mechanism, which culminates in increased stress resistance ultimately leading to an increased lifespan [33]. This cycle, known as mitochondrial hormesis or *mitohormesis*, has been attributed to an active role of ROS as signaling molecules, delivering messages from the mitochondria to other cellular compartments in response to physiological or pathophysiological stimuli. On the whole, the increase in respiration-derived ROS formation should serve as a mild stressor. ROS signals are able to induce conserved downstream processes in the nucleus (such as activation of specific oxidative stress-sensitive MAP-kinase cascades and redox-sensitive transcription factors) that culminate in an overall adaptive response represented by an improvement in the antioxidant response to subsequent stress, delaying age-related changes and finally promoting longevity [33]. In accordance with the hormesis concept, a biphasic behavior was observed in *C. elegans*: low doses of ROS obtained by hyperbaric experimental conditions or hypothermia exerterted a positive effect, whereas higher doses were unquestionably detrimental ([33] and references therein).

## 2. Role of Genetic Variability in Oxidative Stress Response for Healthy Aging and Longevity

In order to develop strategies to maintain oxidative stress at moderate levels, it is necessary to identify the risk factors for the oxidative stress response in the aging organism. Studies suggest that both genetic factors and modifiable lifestyle habits have a major impact on the oxidative stress response. Although it is difficult to estimate the relative contribution of genes and lifestyle in promoting an efficient stress response in cells, studies in experimental organisms and in exceptionally long-lived individuals have attempted to identify the genetic factors modulating the oxidative stress response.

The organism’s stress response is one of the most evolutionary conserved pathways involved in aging and determination of longevity found in animal and human models, together with nutritional sensing and DNA repair/maintenance [34–37]. Genetic screening in model organisms has demonstrated how longevity could be promoted through the manipulation of the response to induced levels of oxidative stress [38]. Mammals also share similar mechanisms, where increased resistance to oxidative stress represents one of the key mechanisms extending lifespan in mutant mice [39].

In humans, the search for a genetic component predisposing to exceptional longevity, *i.e.*, the ability of an individual to extend lifespan over the limits typical of its species, can be carried out by epidemiological studies. Heritability studies in twin cohorts have reported that genetic factors can account for approximately 25% of the variation in human lifespan [40], an effect thought to be minimal before age 60 but most pronounced from 85 years of age and onwards [41]. This evidence prompted research on exceptionally long-lived individuals to uncover the determinants of human lifespan, using cross-sectional and/or longitudinal studies in long-lived and very long-lived cohorts, including “centenarians” [42]. Centenarians represent a very special group of subjects, distinctive from an immune [43], endocrine [44,45] and metabolic [46] standpoint from aged subjects (*i.e.*, those over 65, who are not long-lived according to population specific life-expectancy [47]). Data from centenarian studies suggested that a deep remodeling of important physiological networks operate at extreme age, mainly directed by a genetic predisposition [42]. According to these remodeling theories of aging, long life is the result of a continuous adaptation of the body to deleterious changes over time, and long-lived subjects are endowed with genetic variants allowing them to optimize these cellular responses and better deal with environmental and internal stressors. Evidence in Okinawan centenarians, obtained by measuring the level of lipid peroxide and tocopherol (Vitamin E) in plasma, reported a lower degree of oxidative stress in healthy centenarians (*i.e.*, free of the major age-related diseases) compared to aged subjects [48], which could be due to nutritional factors [49] but also to a specific genetic background. As reported in Table 1, genetic variants affecting anti-oxidant defense mechanisms have been demonstrated to be positively or negatively associated with human longevity in at least one association study [50–97].

Among the anti-oxidant enzymes, a major role in longevity seems to be attributed to genes *SOD2* and *GPX*, for which there are over 80 publications regarding age-related diseases associated with elevated levels of oxidative stress, ranging from cancers to CVD, diabetes and nephropathies ([98] and references therein), supporting the role of genetic variations in the regulation of the oxidative stress response at old age. Included in the same stress response pathway could also be those genes coding for the telomere maintenance pathway, mainly TERT (telomerase reverse transcriptase) and TERC (telomerase RNA component) [86,87], considered that oxidative stress accelerates telomere loss, while telomere protection was found to be associated with CR in the mouse [99]. Some genes involved in the stress response pathway, like *HSPA1A* (Heat Shock Protein A1A) or *PON1* (Paraoxonase 1), showed sex-specific effects, confirming the evidence that the gene networks modulating survival at advanced age in good health show an age- and sex- specific trend [47]. On the other hand, evidence has indicated that the lower degree of oxidative stress in females can depend on the antioxidant role of estrogens, and the consequential up-regulation of longevity-related genes via the estrogen receptor, MAPK activation and NFkB signaling pathways [100].

It is worth noting from the genes reported in Table 1, that the stress response pathway is an integrated network of molecular activities, ranging from immunity to inflammatory regulation and activation, to glucose homeostasis and mitochondrial metabolism. This concept is in line with evidence from recent studies on biological parameters characterizing human longevity suggesting that aging is associated with a loss of complexity in the dynamics of multiple control systems. This loss of control may reduce the ability to adapt to stress, leading to a state of impaired homeostasis, vulnerability to internal and external stressors and ultimately increased frailty [29]. On the contrary, the healthy oldest old (*i.e.*, long-lived healthy individuals free from the major age-related diseases) should be able to maintain a higher level of integration among the different physiological pathways operating within the cell, and thus interacting more successfully with stressors [101]. We have not reported negative evidence, although it is clear that some of the genes listed were extensively investigated in different populations with contrasting results, with only involvement of the *APOE* gene in human longevity being consistently replicated to date [102].

### Mitochondrial DNA Variability, Oxidative Stress and Aging: A Complex Interaction

Mitochondrial genetic variability, both germline (*i.e.*, mitochondrial haplogroups) and somatic, influences the stress response and is associated with human aging/longevity [88–96]. In particular, an association with longevity has been found for the J haplogroup in Northern Italians, Finnish and Irish long-lived subjects [90–93], while haplogroups F and D4 were respectively found in Japanese and Chinese populations [94–96]. Moreover, the coordination of mitochondrial variability with nuclear genetics has been found to have an important role in the genetic susceptibility to human longevity and aging [103]. In particular, by analyzing the distribution of mtDNA inherited variants with respect to nuclear variability in candidate longevity genes, our group observed an over-representation of the U haplogroup in centenarians carrying a *THO* (Tyrosine Hydroxylase) genotype considered unfavorable to longevity [104]. Instead, a compensatory role for mtDNA K and U haplogroups favorable for longevity was also demonstrated in sporadic AD patients by our same group with the predisposing APOE4 alleles [105,106], supporting the hypothesis that some human aging traits and diseases involve specific interactions between mitochondrial and nuclear DNA.

At the molecular level, a strict coordination between nuclear and mitochondrial genomes is necessary to ensure the biosynthesis and functional activity of mitochondria, in both physiological and pathological conditions [107]. In normal conditions, signals from the nucleus to mitochondrion are essential for maintaining an adequate mitochondrial structure and function, the so-called *anterograde response*. Several nuclear-encoded transcription factors and co-activators modulate mitochondrial replication and transcription. Among these are transcription factors which bind to the promoter regions of mtDNA, like the mitochondrial Transcription Factor A (Tfam) and B (mtTFB), enhancing the rate of transcription initiation of mtDNA genes and mitochondrial biogenesis [107,108]; while others, like the Nuclear Respiratory Factors NRF-1 and NRF-2 and the peroxisome proliferator-activated receptor-γ coactivator-1 (PGC-1) family coactivators (PGC-1α, PGC-1α, and related coactivator PRC), act predominantly on nuclear genes, whose products are required for respiratory chain expression and biological function [109,110].

On the other hand, when the stress response is insufficient for counteracting the intracellular impairment and damage occurring in mitochondria, pro-survival or pro-death pathways can be triggered directly by the mitochondria to the nucleus [111]. As a consequence, compensatory signaling which triggers apoptotic or necrotic cell death is activated, in order to balance mitochondrial dysfunctions and avoid further impairments of the whole intracellular metabolism [112–115]. This cellular response, named the *retrograde response*, changes the functional state of mitochondria and results in wide-ranging modifications in nuclear gene expression and consequently cell viability, and has been indicated as an important determinant of the extension of yeast lifespan. In particular, in this animal model, the age-related accumulation of Extrachromosomal Ribosomal DNA Circles (ERCs) and their deleterious effects are mitigated by the retrograde response, with a consequent increase of lifespan [112].

In humans, the experimental demonstration of the existence of a nuclear-mitochondrial cross-talk, and of its importance for the modulation of cellular functionality, is the *in vitro* model of cytoplasmic hybrids also known as cybrids. Cybrid cell lines, first described by King and Attardi [116], are cells that are engineered to share the same nuclear genome but with different mtDNA. They are generated *in vitro* by repopulating mtDNA-null cells (Rho0 cells) with enucleated cells (often platelets) harboring a particular type of mtDNA molecule. Using this experimental approach it is possible to clarify the influences of mtDNA variability on important cellular processes involving the mitochondrion-nucleus cross-talk. In particular, in cybrids with different mitochondrial genomes, cell viability, intracellular calcium dynamics, mtDNA copy number, mitochondrial ROS production, the expression levels of stress responder nuclear-encoded genes, including some cytokines, *HSP60* and *HSP75* as well as *SIRT3* (sirtuin 3, silent mating type information regulation 2 homolog 3), a mitochondrial deacetylase involved in cell metabolism, have been demonstrated to be dependent on the interaction between nuclear and mitochondrial variability [117–120]. Thus, molecular studies support population association studies suggesting an important role of nuclear-mitochondrial dynamics in the genetic determinants of the stress response.

## 3. Role of Lifestyle in the Oxidative Stress Response for Healthy Aging and Longevity

Among lifestyle factors having a major impact on the whole organism oxidative stress response, we can consider impaired nutrition, reduced physical activity, alcohol consumption, and cigarette smoking, which many reports claim as major contributors to the failure of systemic homeostasis, especially if persisting for a long part of the individual’s life. Physical activity and diet, in particular, have been suggested to seriously influence the oxidative stress response in humans, tipping the balance of oxidative burden/antioxidant response to one side or the other.

### 3.1. Physical Activity

A common marker of aging is the progressive decline in physical activity levels, shared by a wide range of species, ranging from the *Caenorhabditis elegans* worm [121] to humans. In humans, physical inactivity has major metabolic consequences, due to a functional decline in of multiple organ systems, which ultimately leads to increased incidence and mortality from diseases such as type II diabetes mellitus, neurodegenerative diseases, cancer, and cardiovascular disease [122]. In contrast, regular physical activity is able to improve metabolic status and insulin sensitivity, as well as modulate age-related changes in weight and body composition in older individuals (*i.e.*, by increasing lean mass and reducing adipose tissue) [123,124]. Moreover, physical activity attenuates several age-related diseases, such as type II diabetes [125], cancer [126], hypertension [127], and osteoporosis [128], and is inversely correlated with mortality [129]. In addition, physical activity reduces blood pressure in hypertensive patients [130], improves the serum lipid profile with an average reduction of 3.7% in triglyceride and 5% in low-density lipoprotein (LDL)-cholesterol levels, and a 4.6% increase of HDL-cholesterol levels [131]. Experimentally, the positive effect of physical activity on maximum life span has been documented in aged rats, where a moderate prolonged exercise was able to induce an increase in SIRT1 activity, suggesting that the sirtuins pathway can be involved in counteracting age-related dysfunctions; at least in those related to muscle activity [132]. Physical activity (especially if begun in mid-life), quitting cigarette smoking, maintaining normal blood pressure, and avoiding obesity are independently associated with overall mortality [133]. Benefits for the maintenance of an optimal health status and the prevention or management of chronic diseases in physically active older people can derive from exercise-induced adaptations of the cellular antioxidant defense systems [134], as demonstrated by the higher serum levels of antioxidants associated with higher strength and physical performance [135–137].

Physical activity has positive effects not only on muscle health but also on the mental health of the elderly by contributing to their well-being and quality of life. The benefits of physical activity on brain aging and neurodegeration is particularly important in our “aging society”, where an increasing number of people are affected by the distressing condition of cognitive aging, which starts with stereotypical structural and neuro-physiological changes, and can determine variable degrees of cognitive decline.

Multiple lines of evidence suggest that progressive oxidative damage is a conserved, and represents a central mechanism of age-related cognitive decline [138] driven by the fact that the brain utilizes 20% more oxygen than other tissues that also undergo mitochondrial respiration, increasing the potential for ROS exposure. The main consequence of oxidative stress/damage in the brain is the accumulation of damaged cellular components, mainly at the level of membrane proteins and lipids.

Regular physical activity seems to protect against brain damage in different anatomic locations (hippocampus, motor cortex, brain stems, cerebellum), by reducing neuronal impairment and promoting the recovery of motor performance, as well as by stimulating vascular mechanisms in the central nervous system [139]. In mice, these effects are accompanied by an increase in IGF1, a hormone with neurotrophic effects, and in BDNF (Brain-Derived Neurotrophic Factor), a protein reported to modulate synaptic plasticity in the adult brain [140]. Muscle exercise increases the amount of IGF1 mRNA and increases the uptake of IGF1 circulating in the brain [140]. In old mice subjected to a training protocol, there is 50% reversal in the neuronal loss in the hippocampus compared to sedentary mice controls of the same age [141]. In humans, elderly people who have engaged in regular physical activity during their lifetime suffer less loss of brain tissue than sedentary subjects and have better cognitive performance [142].

At this point, defining the levels of physical activity that could be useful for delaying health risk becomes a significant, yet difficult issue to be addressed. According to the WHO (World Health Organization) definition, physical activity is “any bodily movement produced by the skeletal muscles which results in energy expenditure above resting level” [143]. However, the quantification of physical activity is highly debated among general practitioners and epidemiologists, due to the difficulty of measuring the levels of intensity, frequency and duration required, as well as establishing a threshold to be reached in order to curb mortality. However, it has been documented that an energy expenditure of 1000 to 1700 kcal per week is associated with a 30% reduction of mortality risk [143]. This is particularly true for performances close to the WHO recommended activity for adults over 65 years, not suffering from chronic diseases, and corresponds to moderate intensity exercise (from 2.5 to 5 h per week or 30–60 min over five days each week), similar to that of young adults. Physical activity of moderate intensity is considered brisk walking, carrying out routine activities in everyday life such as housework, which also includes lifting small weights.

Beyond having positive effects at the molecular level, moderate physical activity contributes to subjective well-being and perceived health status, improving the overall quality of life. While endurance training induces adaptation to the stimulus and increases antioxidant ability, it is important to underline that data from the literature also suggest that acute exercise produces more of a disadvantage than an advantage at old age. This affirmation is mainly due to the fact that intensive physical activity leads to risk of fracture, but is also related to a documented increase in oxidative stress levels following acute bouts of intermittent or anaerobic exercise [144]. The muscle’s adaptation to physical training is always achieved at the cost of a major metabolic stress, in younger as well as in older organisms. However, as the individual ages, intracellular events or *intrinsic factors* can lead to increased oxidative injury during exercise; these include altered biochemical cell structure, increased fragility of muscles associated with transient hypoxia, and re-oxygenation, reduced cell proliferation and protein synthesis that limit antioxidant defense and repair capacity [145]. Acute exercise has been found to generate a higher oxidative damage in muscles in aged mice and men compared to younger subjects [146,147]. Furthermore, there is a reduction in muscle repair and regeneration capacity at old age, as well as a general decline of muscle mitochondrial oxidative capacity [145]. Concurrently, acute exercise is accompanied by a pro-inflammatory response that in many aspects is similar to that induced by infection and sepsis [148]. Nevertheless, the equilibrium between free radical production and antioxidant defense induced by physical exercise in the elderly may be more unstable than in younger subjects, as demonstrated by the higher rate of oxidative stress occurring in older persons [48,49]. If not counterbalanced by a proper dietary antioxidant intake (*extrinsic factor*) and possibly burdened by co-morbidities, *i.e.*, concurrent pathologic conditions which could exacerbate the risk, the balance of oxidants and antioxidants becomes even more delicate with advancing age, potentially enhancing the accrual of cellular oxidative damage.

Ultimately, intrinsic aging may be considered an insufficient ability to respond to endogenous ROS signals, whilst moderate physical activity should have a positive effect on metabolic health by increasing stress resistance over time and thus promoting healthy aging. Balancing negative and positive effects, it can be stated that moderate levels of oxidative stress from physical exercise, not considering the level of endogenous antioxidants, are preventive mechanisms for the organism in order to adapt and reach a new level of hormesis, which can prevent age-dependent sarcopenia and dementia.

### 3.2. Dietary Intake of Antioxidants

The Mediterranean diet and red wine consumption, rich in antioxidants like resveratrol, have been shown to have protective effects against oxidative damage. At the systemic level, their combination was demonstrated to raise plasma Vitamin C and beta-carotene, plasma and urinary polyphenols and the concentration of red blood cells [149]. In general, people who consume large amounts of fruits and vegetables have a lower incidence of CVD, stroke and tumours and it has been proposed that micro nourishments with antioxidant activity could be responsible for the reduction of chronic diseases [150]. Consistently, a diet rich in vegetables and in natural antioxidants has been found to be preferred by long-lived individuals.

Nutritional assessment of Southern Italian centenarians, particularly coming from rural areas where there is a concentration of centenarians 4.32-fold higher than the national average (2.4/10.000) and a female/male ratio of 1:1 (national ratio 4.5:1), demonstrated a prevalence of the Mediterranean diet among long-lived individuals [151]. This nutritional regimen seems to be organized into three meals, and includes reduced quantities of carbohydrates, saturated fat, red meat, refined meats and sweets, and a high intake of fruit and vegetables, whole grains, as well as a large consumption of olive oil and a moderate use of red wine. Beneficial effects of olive oil are due to the presence of a higher proportion of monounsaturated fatty acids, namely oleic acid, while those of red wine are attributed to the presence of polyphenols with antioxidant properties able to buffer the increase of oxidative stress following the ingestion of other nutrients [152]. The regular consumption of fruits, vegetables, whole grains, and other plant foods has been negatively correlated with the risk of development of chronic diseases. Fruit and vegetables are rich in phytochemicals that function as antioxidants, supply vitamins and minerals to the diet and are sources of phytoestrogens, and anti-inflammatory agents.

In addition to the Mediterranean diet, nutritional studies carried out in the Asiatic population suggest that the traditional Okinawan diet, with its high intake of green leafy and yellow root vegetables, sweet potatoes as a dietary staple, and soy as a principle protein, supplemented by small amounts of fish and meat, may be a significant advantage in achieving their exceptionally long life expectancy, thanks to a particularly high amount of antioxidant vitamins [153]. A very important antioxidant role has been recognized for soybeans, a legume with a higher protein content than other plants, recognized for its ability to stimulate the immune system and reduce LDL cholesterol. Its antioxidant capacity has been attributed to a large presence of phenolic acids (like caffeic acid, coumaric acid, *etc.*) and genistein, an isoflavone and phytoestrogen extensively studied in relation to cancer risk. Genistein soy-derived intake has been shown to increase activity of antioxidant enzymes, including SOD, GPX, CAT, and GSR (glutation reductase) [154].

What are the antioxidant properties of vitamins and minerals provided by nutrition? Vitamin C is the major hydrophilic antioxidant and a powerful inhibitor of lipid peroxidation. In membranes, this molecule rapidly reduces α-tocopheroxyl radicals and LDL to regenerate α-tocopherol and inhibit propagation of free radicals [155]. Vitamin E is a lipid-soluble vitamin found in cell membranes and circulating lipoproteins. Its antioxidant function is strongly supported by regeneration promoted by Vitamin C [156] and is thought to prevent atherosclerosis through inhibition of oxidative modification of LDLs [157,158]. Vitamin A belongs to a group of unsaturated hydrocarbons, with multiple functions in growth and development, maintenance of the immune system and good vision. It can be found in two main lipid-soluble forms, retinol or retynil esters and carotenes. The most well-known β-carotene is a potent antioxidant able to quench singlet oxygen [159] and reduce lipid peroxidation [160]; its depletion, as well as that of α-carotene, β-cryptoxanthin, lycopene, and lutein/zeaxanthin, has been associated with atherosclerosis [161], cardiovascular disease [162], sarcopenia [134], and mortality [133]. More generally, Vitamin C, Vitamin E, and carotenoids have shown to synergistically counteract lipid peroxidation [163].

A powerful role of natural antioxidants, although not present from dietary intake, has been recognized for melatonin, a mammalian hormone synthesized from serotonin, mainly in the pineal gland, where this substance contributes to the reduction of oxidative damage [164,165]. Melatonin exerts its antioxidant capacity by stimulating the expression and activity of glutathione peroxidase, superoxide dismutase and inhibiting that of nitric oxid synthase [166].

Behind this general classification, a strict definition of some substances as pro- or antioxidants is somewhat subtle: it is known that trace elements with antioxidant properties, such as copper, zinc, selenium, and molybdenum, may become strongly pro-oxidant both *in vivo* and *in vitro* as a consequence of their physical properties. They are involved in many biochemical processes supporting life, such as cellular respiration, cellular utilization of oxygen, DNA and RNA reproduction, maintenance of cell membrane integrity, and sequestration of free radicals [167].

In addition to having a diet rich in vegetables, people who live longer have a low caloric regimen, with an average daily calorie intake of about 77% with respect that recommended for their gender. Popular studies on this topic were carried out in both Okinawan and Askhenazi centenarians [168,169], in these populations a low calorie intake was reported in exceptionally long-lived individuals, which mimics the effects of CR in animal models. In humans, a low caloric regime is able to delay many diseases associated with aging including cancer, diabetes, atherosclerosis, cardiovascular and neurodegenerative diseases. Also relevant is the possible impact of a caloric restricted nutrition on brain aging, considered that a three-month period of CR in healthy aged humans has been found to be sufficient to improve verbal memory by approximately 20% [170]. This effect is similar to the beneficial effects of CR on age-dependent impairment of learning and memory in rodents [171] and it is in line with the widely documented preventive effects of CR on age-dependent gene expression changes in the mouse brain [172] and on the reduction of age-related brain atrophy in rhesus macaques [173].

Overall, a well-balanced and healthy eating plan, with fewer calorie dishes, rich in vegetables and fruits but low in animal fats, red meat and carbohydrates, can allow the long-lived to benefit from antioxidant natural defenses and also to maintain a healthy weight.

### 3.3. Antioxidant Supplementation to Diet

The identification of free radical reactions as promoters of the aging process implies that pharmacological interventions aimed at limiting or inhibiting ROS should be able to reduce the rate of aging-related changes with a consequent reduction of the aging rate and disease pathogenesis. Even if antioxidant supplementation is receiving growing attention and is increasingly adopted in Western countries, supporting evidence is often equivocal and difficult to interpret.

On one hand, beneficial effects of antioxidants as a therapeutic strategy for complex diseases have been reported both in animal models and humans. For neurological disorders, Vitamin E supplementation was demonstrated to attenuate the toxic effects of β-amyloid and improve cognitive performance in rodents [174], moreover an effect on the reduction of neuronal damage and the progression of AD has also been documented in humans [175]. In cardiovascular diseases, long-term treatment with antioxidants (Vitamin C, Vitamin E, coenzyme Q10 and selenium) significantly increased large and small artery elasticity in patients with multiple cardiovascular risk factors. This beneficial vascular effect was associated with an improvement in glucose and lipid metabolism as well as a decrease in blood pressure [176]. In cancer studies, there has been mounting support for using antioxidants during cancer therapy, starting from trials demonstrating that antioxidant supplementation (glutathione, Vitamin A, Vitamin C, Vitamin E, selenium, and beta-carotene) may help increasing survival rates, tumor response, and patient ability to tolerate chemotherapy treatment [177]. Furthermore, limited evidence for a positive effect of dietary antioxidants on the increase of lifespan was obtained in the mouse; zinc supplementation to the diet promoted healthy aging, and in fact, augmenting dietary zinc intake later in life improved survival and increased murine maximum lifespan [178]. Also resveratrol increases lifespan of rodents provided with a high fat diet, reducing cognitive impairment and preventing AD [179]. Lastly, both açai fruit and curcumin have been demonstrated to increase lifespan in Drosophila [180].

In contrast, there are studies in animal models suggesting that antioxidants do not provide health benefit or, when they do, they do not necessarily determine an increased lifespan [181]. Moreover, where benefit is observed, it appears to be achieved, at least in part, via modulation of biological processes such as increases in nitric oxide bioavailability and induction of protective enzymes such as heme oxygenase-1, rather than via inhibition of oxidative processes, decreased inflammation or apoptosis in the vascular endothelium and arterial wall [182].

In humans, antioxidants, synthetic or naturally occurring, were widely proposed as an anti-aging strategy. However, to date no prospective clinical intervention studies have been able to show a positive association between antioxidant supplementation and increased survival [183,184]. Although some reports have suggested that antioxidants may promote cancer growth [185,186] and generally increase the incidence of a number of diseases with adverse effects on human longevity [187]. An example of the controversial health benefits is the supplementation with selenium for cancer prevention. Selenium was found to prevent prostate cancer in the Nutritional Prevention of Cancer Trial (NPC); these results prompted two additional clinical trials: the Selenium and Vitamin E Cancer Prevention Trial (SELECT) and a Phase III trial, which failed to confirm the results of the NPC trial, and not only demonstrated the inefficacy of these dietary supplements in preventing prostate cancer, but also reported a 17% increased risk in men taking the antioxidant compared to those taking the placebo [188].

These apparently contrasting results can be explained by the amount of anti-oxidant supplementation provided and the consequent de-regulation of the signaling pathways induced by ROS. Given that ROS serve as molecular signals of the endogenous defense mechanisms culminating in increased stress resistance and longevity, antioxidant supplements that prevent these ROS signals interfere with the health-promoting and life-span-extending capabilities of diet and physical exercise. It is also plausible that supplementation strategies are less efficient in Western countries. Furthermore the Western diet is characterized by a massive consumption of meat, a lifestyle factor which has been associated with an increased risk of cancer in the Western population, contrary to the reduced incidence of tumors among more vegetarian populations [189,190].

It is also possible that excess intake of trace elements leads to disease and toxicity [191,192], as it was demonstrated for Vitamins A and C, which may become pro-oxidant under defined conditions [193,194]. Acute and chronic effects of Vitamin A toxicity in bone alterations have been documented in large, prospective, observation studies, carried out in Scandinavia and the United States, countries in which the incidence of osteoporosis is high [193]. These studies have found that excess Vitamin A affects bone fragility, probably due to the antagonism at the receptor level of Vitamin A and D with calcium-regulated hormones, such as parathyroid hormone, leading to higher incidence of hip fracture and osteoporosis among people assuming only twice RDA (Recommended Dietary Allowance). As for Vitamin C, many epidemiological data have indicated that it protects cells from oxidative DNA damage, thereby blocking carcinogenesis, and is associated with a decreased incidence of cancer; however, other studies have indicated that a dietary amount of Vitamin C (200 mg/d) can induce the decomposition of lipid hydroperoxides producing endogenous genotoxins [194]. It has been proposed that this pro-oxidant behavior may depend on the presence of transition metal ions or alkali, such as ferric ions. It must be pointed however that all these data have been obtained from *in vitro* studies, and it is highly questioned whether they can represent a real risk for the human body, considered the limited concentrations of vitamins *in vivo* (due to rapid protein uptake) and to the presence of antioxidant systems to counteract their negative effects.

Thus, it is clear that a fine balance of all these molecules is essential for obtaining real health benefits. Furthermore, these benefits can be better acquired by a proper dietary consumption of antioxidants than by pharmacological supplementation, probably due to the concomitant presence in fruit and vegetables of vitamins and phenolic phytochemicals such as flavonoids, with a strong antioxidant and anticarcinogenetic effects.

## 4. Conclusions

The oxidative stress pathway represents one of the crucial mechanisms linked to human aging and longevity along the evolutionary scale, extending from animal models to humans. The fine regulation of the stress response is crucial for healthy aging considered that an unbalanced stress response can lead to direct cell and tissue impairment and may represent the first step in molecular pathogenesis; while the activation of the oxidative stress pathway can also trigger the onset of disease processes, as suggested for neurological diseases whereby an unbalanced oxidative stress response contributes to neuronal cell death.

From the data reported here it is clear that both genetic background and lifestyle factors can delay or retard deleterious processes involved in the oxidative stress response linked to aging. However the specific endogenous cellular defense mechanisms involved are still unknown, and even fewer data are available regarding the variability of the stress response in different individuals and populations. In fact, although the oxidative stress pathway has been extensively characterized from a biochemical and molecular standpoint, thanks to experimental models and knock-out approaches, population-association studies investigating the role of genetic variability in genes coding for proteins belonging to the oxidative stress cascade, such as key point regulators NFkB or MAP kinases, are still scarce and could be useful for a deeper knowledge of the genetic factors required to provide a more efficient stress response. Besides a favorable genetic constitution, which may predispose an individual to an efficient anti-oxidant defense, adopting a favorable life-style during the full span of the individual’s life may reduce the oxidative stress load and increase antioxidant abilities. In particular, regular physical exercise can be a natural antioxidant and anti-inflammatory strategy for preventing the evolution and complications of age-related diseases such as diabetes and CVD, in addition to maintaining physical capacity, preserving autonomy and improving quality of life during aging, possibly increasing the individual’s chances of achieving longevity.

Finally, considering the contrasting results reported by antioxidant supplementation therapy in the treatment of age-related chronic diseases, further inventigation into the mechanisms controlling the individual’s response to oxidative stress may stimulate the development of pharmaceutical agents able to modulate endogenous cellular defense mechanisms. This innovative approach is underway for diseases causing chronic tissue damage, such as neurodegenerative syndromes [195]. Especially for aged people suffering from different pathologies attributed to oxidative stress burden, further benefit could be derived from the study of gene modulators in longevity-associated pathways, to better address the search for molecules which help individuals to control oxidative stress levels and favor healthy aging [196,197].

**Figure 1:**
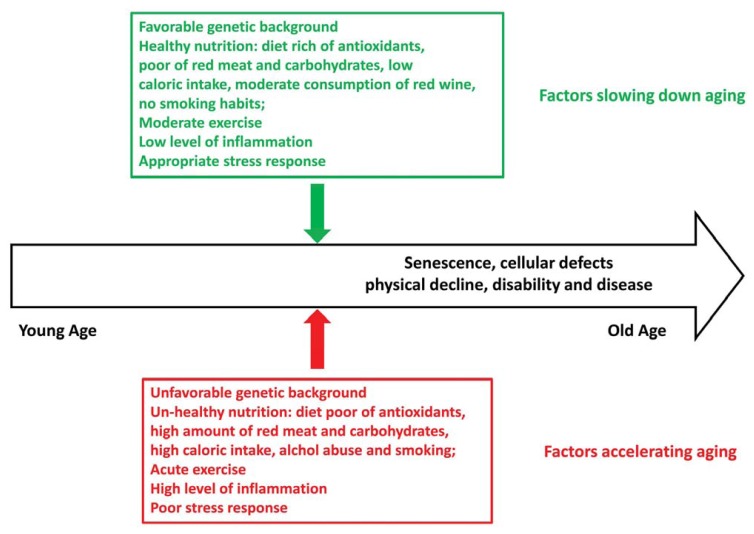
Schematic representation of the different factors modulating the oxidative stress response and influencing healthy aging/longevity. In green is indicated a possible protective profile able to slow down aging and reduce the consequences of impaired homeostasis (senescence, cellular defects, physical decline and disease). On the contrary, a possible detrimental profile for oxidative stress response is indicated in red.

In conclusion, the genetic background undeniably has an important role in the individual’s ability to counteract oxidative stress and to delay the effects of aging. On the other hand, much can be done to minimize the stress load our organism has to endure (Figure 1). In particular, a simple strategy for healthy aging for the elderly population should be to adopt a moderate physically active lifestyle and to ensure an adequate intake of dietary antioxidants to minimize oxidative stress to a basic level. Optimizing nutrition is unquestionably a vital element in avoiding or delaying the onset of age-related diseases and maintaining good health. Social services and policies aimed at improving health status may forsee, as an adjunct to pharmaceutical therapy, social and behavioral interventions which guarantee regular physical activity and social support, in order to reduce the overall chronic stress burden and to provide mental and physical well-being, with the hopes of extending the individual’s chances of living longer and especially in good health.

## Figures and Tables

**Table 1:** Genes for which polymorphisms have been reported successfully associated at least in one study with human longevity.

Gene *	Biological function	Polymorphism	Position of the variation	Direction of association **	References
*Tp53*	Tumour suppressor p53	rs1042522	534 C/G (aa33)	G POSITIVE (Italian; the Netherlands)	[50–52]
*GPX1*	Glutathione peroxidase 1	rs1050450	958 C/T	T POSITIVE (Danish)	[53]
*SOD2*	Manganese superoxide dismutase	rs4880 rs911847	201 T/C (aa16) C/T variation near-gene	C POSITIVE (Danish) POSITIVE (U.S. Caucasian European)	[53] [54]
*NOS1*	Nitric Oxide Synthase1	rs1879417	2087 C/T (aa608)	C NEGATIVE (Italian)	[55]
*NOS2*	Nitric Oxide Synthase 2	rs2297518	−34640 G/A	A NEGATIVE (Italian)	[55]
*HSPA1A*	Heat shock protein	rs1043618	−110 A/T	Allele A NEGATIVE (Italian) AA Genotype NEGATIVE (Danish)	[56] [57]
*HSPA1B*	Heat shock protein	rs1061581	1059 G/A	POSITIVE (Danish)	[57]
*HSPA1L*	Heat shock protein	rs2227956	1661 C/T (aa493)	NEGATIVE (Irish)	[58]
*SIRT1*	Sirtuin protein deacetylase 1	rs7896005 rs3758391	A/G Intron 2 −1138 T/C	G POSITIVE (U.S. Caucasian European) T POSITIVE (The Netherlands)	[59] [60]
*SIRT3*	Sirtuin protein deacetylase 3	rs11555236 rs939915 VNTR (72bp)	477 G/T (aa159) −1409 A/T Intron 5	POSITIVE (Italian) POSITIVE (Italian, German) Allele 2 NEGATIVE (Italian)	[61] [62] [63]
*UCP1*	Uncoupling protein	rs1800592; rs7687015	−3826 A/G; −3737 C/A	A POSITIVE; C POSITIVE (Italian)	[64]
*UCP2*	Uncoupling protein	rs660339	544 C/T (aa55)	POSITIVE (Italian)	[65]
*UCP3*	Uncoupling protein	rs1800849; rs15763 rs11235972	−55 C/T; 118 C/T A/G Intron 3	T POSITIVE (Italian) A NEGATIVE(Danish)	[66] [67]
*SLC25A27 (UCP4)*	Uncoupling protein	rs9472817	C/G Intron 8	G NEGATIVE(Italian)	[65]
*TXNRD1*	Thioredoxin reductase 1	rs10047589	2189 C/T	T POSITIVE (Danish)	[68]
*XDH*	Xanthine dehydrogenase	rs207444	C/T Intron 3	T POSITIVE (Danish)	[68]
*MAP3K7*	Mitogen-activated protein kinase kinase kinase 7	rs282070	C/G Intron 1	POSITIVE (Italian)	[69]
*GSTZ1*	Glutathione S-transferase zeta 1	rs2111699	A/G Intron 1	POSITIVE (Italian)	[69]
*PON1*	Paraoxonase	rs662 rs705379 rs2374983	575 A/G −107 T/C A/G near PON1	POSITIVE (Italian; Irish; German) CC POSITIVE (Italian) POSITIVE (U.S. Caucasian European)	[70–73] [74] [54]
*FOXO1A*	Forkhead box protein O1 A	rs2755209; rs2755213 rs4943794; rs10507486	A/C Intron 1; C/T Intron 1 C/G Intron 1; C/T Intron 1	POSITIVE (Chinese) POSITIVE (U.S. Caucasian European )	[75] [54]
*FOXO3A*	Forkhead box protein O3 A	rs4946936 rs2802292 rs2802288 rs3800231 rs13220810 rs12206094; rs7762395 rs9486902 rs479744; rs9400239 rs2764264; rs13217795	2326 T/C G/T Intron 2 A/G Intron 2 A/G Intron 3 C/T Intron 2 C/T Intron 2; A/G Intron 2 C/T Upstream G/T Downstream; C/T Intron 2 C/T Intron 2; C/T Intron 2	POSITIVE (Chinese) G POSITIVE (Chinese; Japanese; American; Italian) POSITIVE (Italian) POSITIVE (German; Ashkenazi Jewish POSITIVE (German; Danish) POSITIVE (Danish) POSITIVE (Danish) POSITIVE (German; Danish) POSITIVE (Chinese, Danish)	[75] [75–77] [77] [78,79] [78,80] [80] [80] [78,80] [75,80]
*APOE*	Apolipoprotein E	rs429358 (ɛ4) rs7412 (ɛ2)	388 T/C (aa130) 526 C/T (aa176)	NEGATIVE (Italian, Danish, Finnish, French, Japanese) POSITIVE (Italian, Danish, Finnish, French, Japanese)	[81] and references therein
*INS*	Insulin	rs3842755	+286 G/T	POSITIVE (Danish)	[68]
*INSR*	Insulin receptor	rs3745548	A/G Intron 10	POSITIVE (Japanese)	[82]
*IGF1*	Insulin Growth Factor 1	CA repeat (promoter)	Intron 1	POSITIVE (The Netherlands)	[83]
*IGF1R*	Insulin Growth Factor 1 receptor	rs2229765	3179 G/A	A allele POSITIVE (Italian)	[84]
*IGF2*	Insulin Growth Factor 2	rs112276039	490 T/C	A allele POSITIVE (Ashkenazi Jewish)	[85]
*IGF2R*	Insulin Growth Factor 2 receptor	rs9456497	A/G Intron 4	POSITIVE (Danish)	[68]
*IRS1*	Insulin Receptor Substrate 1	rs1801278	2963 G/A	POSITIVE (The Netherlands)	[83]
*GH1*	Growth Hormone 1	rs2665802	+1169 A/T (Intron 4)	T POSITIVE (The Netherlands)	[83]
*GHSR*	Growth Hormone Secretagogue Receptor Type 1	rs572169	520 A/G	POSITIVE (Danish)	[68]
*AKT*	V-Akt Murine Thymoma Viral Oncogene Homolog 1	rs3803304	+19835 G/C	POSITIVE (U.S. Caucasian European; Ashkenazi Jewish)	[79]
*TERC*	Telomerase RNA component	rs3772190 rs12696304	(169500487) C/T 6578 G/C	Allele A POSITIVE (Danish) POSITIVE (Ashkenazi Jewish)	[86] [87]
*TERT*	Telomerase reverse transcriptase	rs2853669; rs2736098, rs33954691; rs2853691 MNS16A VNTR	−245 T/C; 915 G/A; 3039 C/T; +893 A/G Downstream exon 16	POSITIVE (Ashkenazi Jewish) L allele NEGATIVE (Italian)	[87] [88]
*mtDNA*	Mitochondrial DNA	Heteroplasmy Haplogroup J Haplogroup D4 Haplogroup F		High level mtDNA Heteroplasmy POSITIVE (Italian) POSITIVE (Italian, Irish, Finnish) POSITIVE (Japanese) POSITIVE (China)	[88,89] [90–93] [94] [95,96]

* HUGO gene names are reported;

** POSITIVE and NEGATIVE notations refer to positive and negative effect on human longevity, respectively. In brackets, the sample population where the association was observed is reported.
